# Factors Affecting Volume Reduction Velocity for Arteriovenous Malformations After Treatment With Dose-Stage Stereotactic Radiosurgery

**DOI:** 10.3389/fonc.2021.769533

**Published:** 2021-12-20

**Authors:** Xiangyu Meng, Dezhi Gao, Hengwei Jin, Kuanyu Wang, Enmeng Bao, Ali Liu, Youxiang Li, Shibin Sun

**Affiliations:** ^1^ Beijing Neurosurgical Institute, Beijing Tiantan Hospital, Capital Medical University, Beijing, China; ^2^ Department of Gamma-Knife Center, Beijing Neurosurgical Institute, Beijing Tiantan Hospital, Capital Medical University, Beijing, China; ^3^ Department of Interventional Neuroradiology, Beijing Neurosurgical Institute, Beijing Tiantan Hospital, Capital Medical University, Beijing, China

**Keywords:** arteriovenous malformations, dose-stage stereotactic radiosurgery, radiomics, morphologic feature, volume reduction

## Abstract

**Background and Purpose:**

The purpose of this study was to identify morphologic and dosimetric features associated with volume reduction velocity for arteriovenous malformation (AVM) after dose-stage stereotactic radiosurgery (DS-SRS).

**Methods:**

Thirty patients with intracranial AVM were treated with DS fractionated SRS at Beijing Tiantan Hospital from 2011 to 2019. The AVM nidus was automatically segmented from DICOMRT files using the 3D Slicer software. The change in lesion volume was obtained from the decrease in the planning target volume (PTV) between the two treatment sessions. The volume reduction velocity was measured by the change in volume divided by the time interval between treatments. Fourteen morphologic features of AVM prior to treatment were extracted from the PTV using ‘Pyradiomics’ implemented in Python. Along with other dosimetric features, univariate and multivariate analyses were performed to explore predictors of the volume reduction velocity.

**Results:**

Among the 15 male (50.0%) and 15 female (50.0%) patients enrolled in this study, 17 patients (56.7%) initially presented with hemorrhage. The mean treatment interval between the initial and second SRS was 35.73 months. In multivariate analysis, the SurfaceVolumeRatio was the only independent factor associated with the volume reduction velocity (p=0.010, odds ratio=0.720, 95% confidence interval: 0.560–0.925). The area under the curve of this feature for predicting the volume reduction velocity after the initial treatment of DS-SRS was 0.83. (p=0.0018).

**Conclusions:**

The morphologic features correlated well with the volume reduction velocity in patients with intracranial AVM who underwent DS-SRS treatment. The SurfaceVolumeRatio could predict the rate of volume reduction of AVMs after DS-SRS.

## Introduction

Stereotactic radiosurgery (SRS) is gradually becoming a common therapeutic method for intracranial arteriovenous malformations (AVMs). In the treatment of small and moderately sized AVMs, SRS has been demonstrated as a beneficial and low complication-causing treatment during the follow-up period (1, 2). Fractionated stereotactic radiosurgery (FSRS) can be used to avoid complications induced by a single high dose applied to all target lesions for large or high-grade AVMs. Volume-stage (VS) and dose-stage (DS) SRS are multi-session techniques aimed at improving the risk-to-benefit profile for radiotherapy of large AVMs (3–6). The DS strategy was proposed for patients with AVM who have a large planning target volume (PTV); a relatively low dose was converged on the whole lesion for several repeats until the cumulative total dose was applied to the PTV. There was a waiting period of approximately 3–4 years between the two sessions of treatment, which reduce the possibility of radioactive edema caused by excessive PTV. The VS strategy has been proposed for patients with multiple lesions or large volumes with a clear boundary in each encephalic region. VS-SRS divides large volumes into distinct sections, each of which is independently targeted by SRS with intervals until the entire target volume is involved in the PTV.

Many current studies have confirmed that FSRS plays a positive role in the treatment of large or high-grade AVM, either as a stand-alone therapy or as a component of a multimodality management strategy (7). However, the treatment strategy of DS-FSRS remains controversial due to its lower obliteration rate and higher incidence of complications compared with VS-SRS (8, 9). Moreover, which of the patients were more suitable for DS and which were not remains unclear. Research on the choice of therapeutic strategies for patients undergoing fractionated treatment remains insufficient. The purpose of this study was to identify independent factors related to the volume reduction velocity and the indicators that can be used to predict the volume reduction velocity in order to guide the treatment planning of DS-SRS treatment.

## Method

### Patient Selection

This study was approved by the Institutional Ethics Committee of Beijing Tiantan Hospital, and informed consent was obtained from each patient before the procedure. Patients diagnosed with intracranial AVM and treated with DS-SRS at Beijing Tiantan Hospital from January 2011 to December 2019 were retrospectively reviewed. Patients treated with other modalities of therapy during the interval of fractionated sessions were excluded because of the inaccuracy in calculating the volume reduction. Patients who were treated for a single treatment purpose and those who received VS-SRS treatment were excluded. Demographic and radiographic data of each patient were reviewed.

### Treatment Process

The SRS treatment plan was designed based on the consensus of two experienced senior neuroradiologists. When the treatment volume was >10cm^3^, a fractionated treatment process was considered. Although VS-FSRS is reportedly better than DS-FSRS (8, 10), for the nidus close to the important eloquent areas or organs of patients, and in the case that there was no obvious segmentation boundary, and planning VS treatment was difficult, DS treatment was administered to patients, considering the high complications of VS treatment. The treatment procedure began with the placement of a Leksell helmet (G model, Elekta Instrument AB) and stereotactic magnetic resonance imaging (MRI) (1.5-T Magneton Vision, Siemens) was then performed for contouring the target volume and planning distribution of dosage. Digital Subtraction Angiography (DSA) images were used as auxiliary supplementary images for treatment planning. The Leksell Gamma Knife Perfexion machine (Elekta) was used for FSRS with multiple sessions, and all the treatment planning data and stereotactic MRI were preserved in this machine as DICOMRT (Radiotherapy in DICOM) files including PTV and dosage information.

### Calculating Volume Reduction Velocity

The first two fractionated treatment sessions were selected as the original data source to calculate the volume reduction velocity. Treatment DICOMRT files were exported from the Leksell Gamma Knife Perfexion machine and then imported into the 3D Slicer software. With the help of the SlicerRT toolkit, the contouring of the PTV of each session was automatically segmented and preserved as NIFTI files. The PTV for the initial and second treatment sessions were calculated using the software. Then, the volume reduction between the two sessions was calculated by the change in the PTV. The prescription dose, maximum dose, and mean dose of the first treatment were exported as the pre-treatment dosimetric factors using the SlicerRT toolkit. The volume reduction velocity was presented as the volume reduction between the two sessions divided by the time interval.

### Morphological Features of Pre-Treatment Nidus

**Figure 1:**
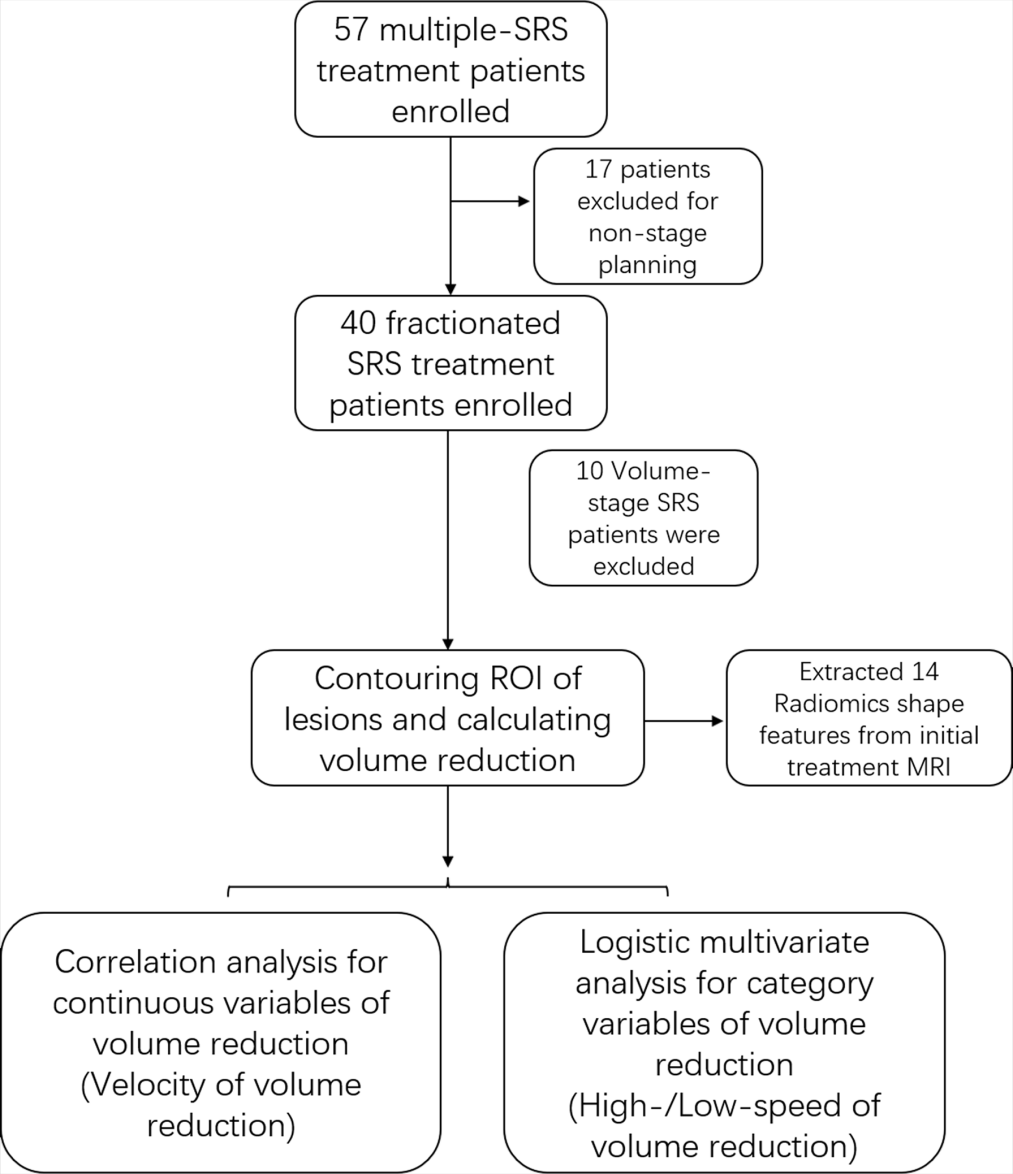
The flowchart illustrated the method and procedure of this study.

The segmentation of the initial treatment MRI was used to extract the morphological features of the AVM nidus. Thirteen shape features were extracted from stereotactic MRI using the ‘Pyradiomics’ package implemented in Python. All these features were extracted from the original image of stereotactic MRI and only 3D shape features were preserved, including VoxelVolume, SurfaceArea, SurfaceVolumeRatio, Sphericity, Maximum3Ddiameter, Maximum2DdiameterSlice, Maximum2DdiameterColumn, Maximum2DdiameterRow, MajorAxis, MinorAxis, LeastAxis, Elongation, and Flatness. A flow chart of the study is shown in **Figure 1**.

### Statistical Analysis

Continuous variables are presented as mean ± SD, and the differences between two groups were compared using Student’s t-test. For categorical variables, data are presented as the number of events followed by relative frequencies (%). A cutoff value of volume reduction velocity was used to classify patients into high-speed and low-speed groups. Univariate analysis was performed to compare the value of each variable between the high-and low-speed groups. Variables with a p value <0.05, were entered into the multivariate logistic analyses to identify the independent predictors of the volume reduction velocity for DS-SRS. A receiver operating characteristic curve was used to analyze the efficiency of features predicting the velocity after fractionated treatment. We performed statistical analysis and plotted the figures using R and GraphPad software.

## Results

### Demographic, Dosimetric, and Morphologic Characteristics Of Patients

Thirty patients (15 males and 15 females) diagnosed with AVM and treated with DS-FSRS were enrolled in our study. The mean age of this cohort was 23.57 years. Among the enrolled patients, 17 presented (56.67%) with hemorrhage, 4 (13.33%) with seizures, 3 (10.00%) with headache, 5 (16.67%) with neurological deficit, and 1 (3.33%)diagnosed accidentally.

The mean prescription dose set on the nidus for the initial treatment was 14.83 Gy (range, 13.00–17.00) with a mean maximum dose of 29.85 Gy (range, 23.60–34.00). The mean PTV dose was measured using the SlicerRT toolkit implemented in the 3D Slicer. The mean PTV dose for the initial treatment was 19.72 Gy (range, 16.32–24.29). The mean PTV was 12.68 cm^3^ for the initial treatment, including 6 patients with nidus <10 cm^3^. Four of the six patients had a mean volume of 6.07 cm^3^, located at the brain stem and thalamus, which could not handle the dose of a single stage treatment. The other two patients also had lesions near the optic chiasm and epidermis, and a single treatment could cause radiation damage. The mean maximum length of the PTV was 3.89 cm. There were 24 nidus (80.00%) evolved in the eloquent area, and 15 nidus (50.00%) drained by deep draining veins. The mean radiosurgery based arteriovenous malformation score (RBAS) was 2.12, and the Spetzler-Martin or Virginia radiosurgery AVM scale (VRAS) is shown in **Table 1**.

**Table 1:** Baseline characteristics of enrolled patients.

Character	NO
Number of patients	30
Gender	
Male	15 (50.00%)
Female	15 (50.00%)
Initial presentations	
Hemorrhage	17 (56.67%)
Epilepsy	4 (13.33%)
Headache	3 (10.00%)
Neurological deficit	5 (16.67%)
No symptom	1 (3.33%)
Location of lesions	
Frontal and temporal lobe	4 (13.33%)
Cerebellum	1 (3.33%)
Basal ganglia and thalamus	5 (16.67%)
Brain stem	7 (23.33%)
Other brain lobe	13 (43.34%)
Eloquent or non-eloquent	
Eloquent	24 (80.00%)
Non-eloquent	6 (20.00%)
Drainage vein	
Deep	15 (50.00%)
Superficial	15 (50.00%)
Spetzler-Martin grade	
I	0
II	4 (13.33%)
III	13 (43.34%)
IV	7 (23.33%)
V	0
VI	6 (20.00%)
Radiosurgery-Based AVM Score (RBAS, mean)	2.12
Virginia Radiosurgery AVM Score (VRAS)	
I	0
II	3 (10.00%)
III	13 (43.34%)
IV	14 (46.66%)

### Univariate and Multivariate Analysis for Time-Stage SRS

**Figure 2:**
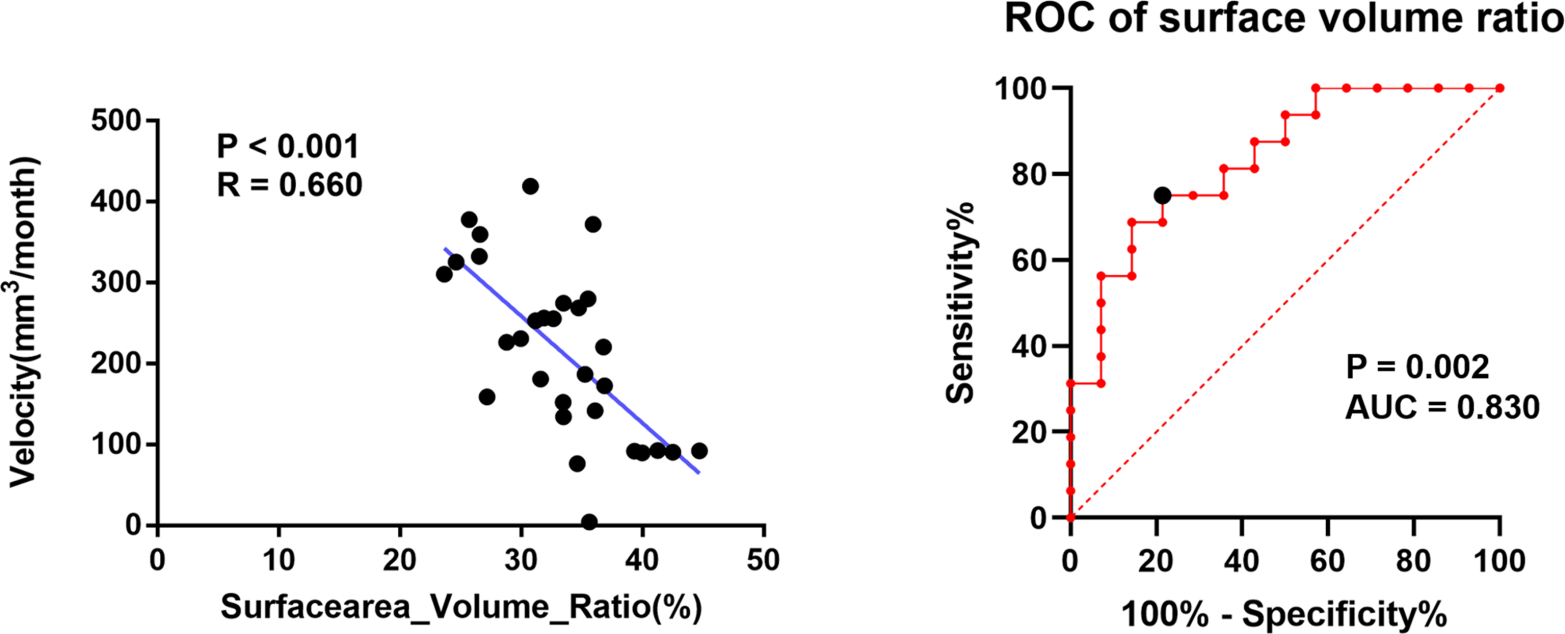
The relationship of SurfaceVolumeRatio with the volume reduction velocity after initial dose-stage SRS treatment. Left figure showed a strong correlation in Pearson Correlation analysis of the SurfaceVolumeRatio and the volume reduction velocity (p<0.001, R=0.66); Right figure showed the ROC curve of using SurfaceVolumeRatio to determine the nidus into high-/low-speed groups in volume reduction separated by a cutoff derived from the volume reduction velocity. (p=0.002, AUC=0.83) The best cutoff value for this feature was 0.335 to classify the high-/low-speed of volume reduction, with a sensitivity and specificity of 0.75 and 0.79, respectively.

After initial treatment, the mean volume reduction during the two sessions of DS-SRS was 8.11 cm^3^ and the mean interval of treatment time was 40.13 months. The mean volume reduction velocity was 0.21 cm^3^/month for all cohorts. The volume reduction velocity was compared between patients with high (>16 Gy) and low (<14 Gy) prescription dose; no statistical difference was found between the two groups (p=0.102). The mean volume reduction velocity was used as the cutoff to distinguish between the high-speed and low-speed groups of volume reduction. Univariate analysis was used to identify the factors predicting the volume reduction velocity. The results from MajorAxisLength, Maximum2DDiameterColumn, Maximum2DDiameterSlice, Maximum3DDiameter, SurfaceArea, SurfaceVolumeRatio, and volume of initial PTV were morphological features that were significantly different between the high-and low-speed groups (p<0.05). RBAS scores of initial FSRS were statistically different between the two groups (p<0.05). In multivariate analysis, the factor associated with volume reduction velocity for initial DS-FSRS treatment was the SurfaceVolumeRatio [p=0.01, odds ratio (OR) = 0.720, 95% confidence interval (CI): 0.560–0.925, (**Table 2**)]. The area under the curve of ROC using SurfaceVolumeRatio to predict the high-/low-speed of volume reduction after DS-FSRS was 0.83 (p=0.002). The best cutoff value for this feature was 0.335 to classify the high-/low-speed of volume reduction, with a sensitivity and specificity of 0.75 and 0.79, respectively. This feature also showed a strong correlation with the volume reduction velocity according to the Pearson correlation test. (p<0.001, R=0.66, **Figure 2**).

**Table 2:** Dosimetric and morphologic features for Dose-stage SRS patients.

Factors	Value for all patients (mean ± SD)	Value for high-speed group (mean ± SD)	Value for low-speed group (mean ± SD)	Univariate analysis	Multivariate analysis	
				P value	P value	95% CI
Presicription dose (Gy)	14.83 ± 1.08	14.56 ± 1.15	15.14 ± 0.93	0.14	–	–
Maximum dose (Gy)	29.85 ± 2.30	29.64 ± 2.70	30.08 ± 1.81	0.61	–	–
Mean dose (Gy)	19.72 ± 1.58	19.42 ± 1.64	20.06 ± 1.50	0.28	–	–
original_shape_Elongation	0.71 ± 0.14	0.68 ± 0.15	0.74 ± 0.12	0.22	–	–
original_shape_Flatness	0.54 ± 0.12	0.52 ± 0.12	0.56 ± 0.12	0.33	–	–
original_shape_LeastAxisLength (mm)	20.12 ± 3.99	21.19 ± 3.79	18.89 ± 3.99	0.12	–	–
original_shape_MajorAxisLength (mm)	38.89 ± 9.18	42.32 ± 7.98	34.97 ± 9.13	0.028*	0.33	0.946-1.173
original_shape_Maximum2DDiameterColumn (mm)	38.38 ± 6.63	41.05 ± 5.70	35.34 ± 6.47	0.017*	0.35	0.871-1.332
original_shape_Maximum2DDiameterRow (mm)	39.70 ± 7.42	41.60 ± 4.91	37.53 ± 9.26	0.16		
original_shape_Maximum2DDiameterSlice (mm)	36.95 ± 8.16	39.84 ± 7.57	33.65 ± 7.76	0.036*	0.63	0.898-1.221
original_shape_Maximum3DDiameter (mm)	45.16 ± 9.03	48.85 ± 6.84	40.94 ± 9.61	0.017*	0.33	0.635-1.435
original_shape_MinorAxisLength (mm)	26.65 ± 4.16	27.86 ± 3.45	25.26 ± 4.59	0.096	0.88	0.651-1.529
original_shape_Sphericity	0.65 ± 0.05	0.66 ± 0.04	0.65 ± 0.07	0.63	–	–
original_shape_SurfaceArea (cm^2^)	39.78 ± 11.59	44.64 ± 9.17	34.23 ± 11.85	0.014*	0.71	0.995-1.006
original_shape_SurfaceVolumeRatio	0.33 ± 0.05	0.31 ± 0.04	0.37 ± 0.05	0.001*	0.010*	0.560-0.925
original_shape_VoxelVolume (cm^3^)	12.68 ± 5.47	15.24 ± 5.20	9.75 ± 4.26	0.004*	0.86	0.999-1.000
						

*The cutoff value for velocity of volume reduction was 0.21 cm3/month in Time-stage SRS patients.

## Discussions

The treatment approach for large AVMs remains controversial. FSRS has been the optimal choice for patients with specific AVM patients. In previous studies, a direct comparison between the volume and time stages was rarely observed. A systematic review and meta-analysis showed that the VS-SRS treatment modality always presents a better prognosis for both obliteration rate and partial obliteration than DS treatment (9). Ilyas et al. (10) reported that the complication rate was significantly higher than that of DS-SRS with a higher obliteration rate of VS-SRS treatment. However, in these studies, which patients should be treated with VS-SRS or DS-SRS was not clarified. In our study, while we did not focus on the comparison of different fractional modalities, the volume reduction velocity for the nidus that underwent DS-SRS treatment was calculated, which reflected the response and sensitivity of the AVM nidus to DS-SRS treatment, and could preliminarily indicate the trend of reduction of nidus after initial treatment. Furthermore, the analysis of DS-SRS revealed a tendency of a smaller SurfaceVolumeRatio to predict a higher volume reduction rate. SurfaceVolumeRatio was calculated from the ratio of the surface area to the volume of the segmented mask, with a lower value indicating a more compact (sphere-like) shape of the target nidus involved in our planning target. This result is consistent with our previous treatment experience that a compact large nidus is more suitable for DS-SRS treatment because it is easier for dose planning, and a separated or irregular nidus should be applied for VS-SRS treatment for its clear segments. Therefore, the application of this feature to predict the volume reduction velocity after treatment with DS-SRS is feasible and can be used to assist in the selection of patients for individualized treatment, as a reason to shorten the interval of sessions and lower the probability of severe complications.

**Figure 3:**
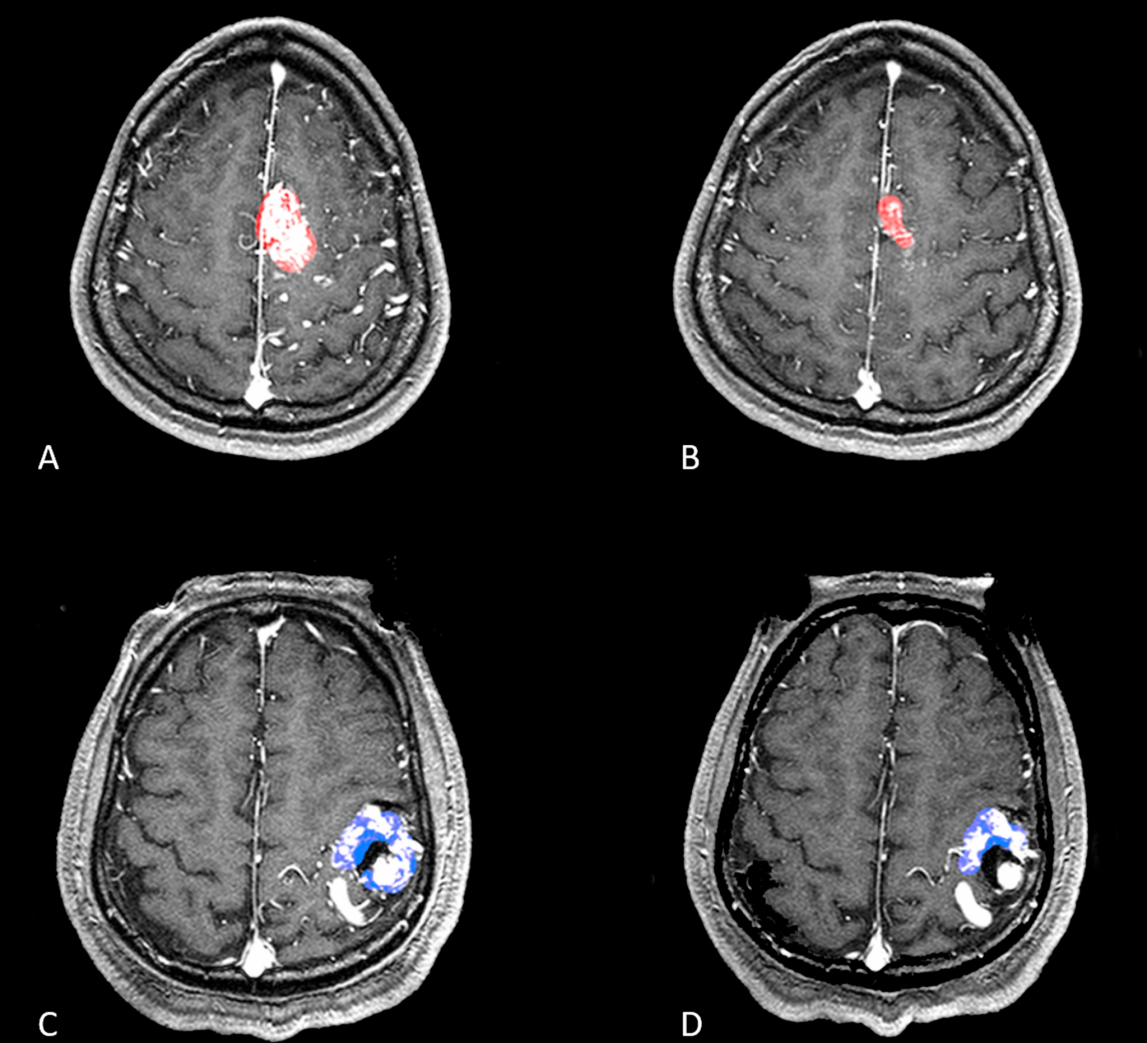
Two patients with a relatively similar volume of nidus but different SurfaceVolumeRatio were shown in **(A–D)**. **(A, B)** A patient with a volume nidus of 11.73ml was applied with a prescription dose of 17 Gy, maximum dose of 34 Gy for dose-stage SRS, and the SurfaceVolumeRatio of this patient was 0.312 which was lower than the best cutoff value in ROC curve. The second treatment was shown in **(B)**, and the mean volume reduction velocity for this patient was 0.253 cm^3^/month. **(C, D)** A patient with a volume nidus of 9.75ml was applied with a prescription dose of 16 Gy, maximum dose of 32 Gy for dose-stage SRS, and the SurfaceVolumeRatio of this patient was 0.447 which was higher than the best cutoff value in ROC curve. The second treatment was shown in **(D)**, and the mean velocity of volume reduction for this patient was 0.092 cm^3^/month.

In this study, more attention was paid to the original morphologic features of the AVM nidus. The shape features of radiomics were extracted to represent the morphological characteristics of the AVM nidus. These features are commonly used in tumors but are rarely present in cerebral vascular diseases (11, 12). A mean volume reduction velocity was first presented as the volume change rate between the two sessions of FSRS treatment. In an analysis of the predictors for the volume reduction velocity after DS-SRS, an independent factor was the morphological features that reflected the regularity of the shape of the mask. Although many features indicating the original morphology of the nidus (PTV) were screened out in the univariate analysis. Only a factor that reflects the degree of regularity of the nidus remains from multivariate analysis. This implies that this characteristic determines how quickly the nidus responds to the DS-SRS treatment (**Figure 3**). The dosimetric features were not significantly different between the high-and low-speed groups in the univariate analysis. Seymour et al. (13) reported that the obliteration rates in diffuse nidus with <17 Gy were particularly poor, with no obliteration in their multi-center research on VS treatment. Apart from this conclusion that the treatment outcome was dosage dependent, the volume reduction velocity was only morphologically dependent on the FSRS in our study. This may be attributed to the relatively uniform dose distributions of all patients receiving the treatment in our center in this study. None of the patients received too high or too low doses. This result indicates that under the dose distribution applied consistently to patients with AVM, using the pre-treatment morphological features can preliminarily predict the tendency of nidus reduction. This can help clinicians to preliminarily screen more suitable patients for DS treatment using morphological features.

The treatment strategy for large AVM lesions still remains elusive, especially in patients with unruptured lesions. The results from the ARUBA trial (A Randomized Trial of Unruptured Brain Arteriovenous Malformations) suggested that conservative treatment is a better modality than other interventional approaches (14). However, such patients tend to have a higher chance of neurological deficits, while some may suffer from excessive tension and anxiety, which may affect their normal life. Therefore, it is necessary to select an appropriate population for interventional therapy. VS-FSRS has been demonstrated to be effective for large-volume AVMs in previous studies. In our study, we only focused on the DS-FSRS, which is called the hypofractionated SRS strategy because of its controversial effectiveness in obliterating the lesions of large AVM. Tam et al. (15) found that the mRBAS score was an independent factor affecting the obliteration rate after hypofractionated SRS in a 5-year follow-up study. None of the AVMs with mRBAS>5.35 were obliterated. This result is consistent with the conclusion of Chen et al. (6) They also found that the irradiated drainage vein volume indexed to AVM volume (volume of drainage vein involved in PTV/AVM volume) correlates well with the increased risks of post-SRS hemorrhage and reduced event-free survival. This result was consistent with the perception of our study that dose level was not a determinant of the volume reduction velocity after hypofractionated treatment, but rather the morphological characteristics of the lesion planned into the PTV. Combined with this study, the patients with lower mRBAS and SurfaceVolumeRatio values may imply the ability to reach obliteration faster and better. Further studies are needed to validate this conclusion in a large prospective cohort study.

The current study had some limitations. First, the cumulative patient sample size was relatively small. Second, the mean PTV was calculated as 12.68 cm3 in the current study, which was smaller than the mean volume presented in previous systematic reviews of 296 patients. Further studies are needed to extend the results of this study to the treatment of large volume DS-FSRS. Finally, this study was a retrospective review, in which the volume reduction velocity was calculated by a mean value that was not precise for the instantaneous speed. In future studies, a prospective study will be needed to calculate the velocity for each patient at the same follow-up end time.

## Conclusions

In our research, we used the mean velocity calculated by the change in volume divided by the interval between sessions to reflect the rate of volume reduction. This rate not only reflects the sensitivity of the nidus to fractionated treatment but also has an impact on the development of treatment and follow-up strategies. The morphologic features correlated well with the volume reduction velocity in intracranial AVMs who underwent FSRS treatment. These features could predict the rate of volume reduction in the AVM after FSRS.

## Data Availability Statement

The raw data supporting the conclusions of this article will be made available by the authors, without undue reservation.

## Ethics Statement

The studies involving human participants were reviewed and approved by the IRB of Beijing Tiantan Hospital, Capital Medical University. Written informed consent to participate in this study was provided by the participants’ legal guardian/next of kin.

## Author Contributions

This study was designed by YL, AL, and SS. Material preparation, data collection and analysis were performed by KW, HJ, and EB. The first draft of the manuscript was written by XM and DG. All authors commented on previous versions of the manuscript. All authors contributed to the article and approved the submitted version.

## Funding

This study has received funding by the Multicenter Clinical Research of Gamma Knife Radiosurgery for The Treatment of Acoustic Neuroma (2019-N-11-37); the Multicenter Clinical Research of Gamma Knife Radiosurgery for The Treatment of Nonfunctional Pituitary Adenoma (2019-N-11-35) and the Registry of Multimodality Treatment for Brain Arteriovenous Malformation in China [HX-A-008(2020)].

## Conflict of Interest

The authors declare that the research was conducted in the absence of any commercial or financial relationships that could be construed as a potential conflict of interest.

## Publisher’s Note

All claims expressed in this article are solely those of the authors and do not necessarily represent those of their affiliated organizations, or those of the publisher, the editors and the reviewers. Any product that may be evaluated in this article, or claim that may be made by its manufacturer, is not guaranteed or endorsed by the publisher.
